# Surface Plasmon Resonance (SPR) for the Binding Kinetics Analysis of Synthetic Cannabinoids: Advancing CB1 Receptor Interaction Studies

**DOI:** 10.3390/ijms26083692

**Published:** 2025-04-14

**Authors:** Xuesong Shi, Lixin Kuai, Deli Xu, Yanling Qiao, Yuanyuan Chen, Bin Di, Peng Xu

**Affiliations:** 1 School of Pharmacy, China Pharmaceutical University, Nanjing 210009, China; shixuesong0109@yeah.net (X.S.); kuailx@163.com (L.K.); 15684549698@163.com (D.X.); qiaoyanling1@126.com (Y.Q.); m13276621173@163.com (Y.C.); 2 China Pharmaceutical University Joint Laboratory on Key Technologies of Narcotics Control, Office of China National Narcotics Control Commission, Beijing 100193, China; 3 Key Laboratory of Drug Monitoring and Control, Drug Intelligence and Forensic Center, Ministry of Public Security, Beijing 100193, China

**Keywords:** Surface Plasmon Resonance technology, synthetic cannabinoids, CB1 receptor affinity

## Abstract

Synthetic cannabinoids (SCs), a class of widely abused new psychoactive substances, are characterized by their structural diversity and rapid evolution. Structure–affinity relationships are crucial for predicting pharmacological effects and potential toxicity. Traditional methods for affinity testing are often complex and less applicable to newly modified compounds. In contrast, Surface Plasmon Resonance (SPR) is a sensitive and label-free technology that detects molecular interactions by measuring refractive index changes on a metallic surface with the advantages of high sensitivity, low sample consumption, and high-throughput capability. In this study, we used SPR to determine the receptor affinity constants of 10 SCs, including some first-reported substances, and analyzed their structure–affinity relationships to validate the method’s reliability. The results showed that (1) indazole-based SCs exhibited stronger CB1 receptor affinity compared to their indole counterparts, (2) the head structure of p-fluorophenyl enhanced affinity relative to 5-fluoropentyl, (3) and the affinity rankings obtained from SPR experiments were consistent with those derived from traditional methods. These results collectively demonstrate the reliability and effectiveness of SPR in assessing CB1 receptor affinity and differentiating affinity differences among structurally similar analogs, with promising application prospects in drug research, particularly in the development and screening of therapeutic agents targeting cannabinoid receptors.

## 1. Introduction

Synthetic cannabinoids (SCs) are a class of synthetic new psychoactive substances (NPSs) designed to produce pharmacological effects by binding to the endogenous cannabinoid receptor CB1 and activating associated signaling pathways, thereby mimicking the in vivo effects of tetrahydrocannabinol (THC), which is the primary active ingredient in natural cannabis. Unlike THC, a partial agonist of the CB1 receptor, most SCs exhibit full agonist activity at the CB1 receptor [1]. Their higher receptor efficacy results in more potent pharmacological effects, including exacerbated toxicities (e.g., neurotoxicity and cardiotoxicity), increased adverse effects, and elevated abuse potential compared to THC [2,3,4], contributing to significant public health risks. Therefore, it is important to study the affinity of SCs for CB1 receptors in order to understand their mechanism of action, predict toxicity levels, and develop antagonists [5].

The skeletal structure of SCs typically comprises four key components: the head, the tail, the parent core, and the linking group [6]. Notably, structural modifications or reconfigurations of any of these components can yield novel variants that retain or even enhance cannabinoid receptor agonistic activity [7]. This structural flexibility has been exploited by illicit manufacturers who continuously modify and synthesize new analogs with enhanced pharmacological properties, including higher receptor affinity, improved blood–brain barrier permeability, as well as stronger toxicity and addiction potential, leading to the rapid proliferation and diversification of SCs on the illegal drug market. SCs are typically classified according to their parent cores [8], with those featuring indoles and their derivatives as the parent nucleus being the most prevalent. First-generation SCs are characterized by indole as the parent core and exemplified by JWH-018 (widely abused in the early 21st century), and subsequently, the second generation (e.g., AM-2201 and PB-22) arose from structural modifications of their head, tail, or linker moieties. In recent years, a novel class of SCs featuring an indazole core has emerged [9], exemplified by “Natasha” [10] and “Twiggy” [11] (whose potent CB1 receptor affinity was confirmed in 2020), demonstrating superior receptor-binding capabilities. The strong structure–activity relationship (SAR) observed in SCs highlights the critical need for a systematic analysis of their structural characteristics and receptor-binding affinities. Investigations into these structure–affinity relationships not only enable the prediction of receptor-binding properties of emerging SCs but also contribute to understanding their pharmacological and toxicological profiles [12], which could significantly support the development of more targeted regulatory measures and evidence-based harm reduction strategies.

The conventional methods for evaluating CB1 receptor affinity, including Radioligand Receptor-Binding Assay (RRA) [13], Competitive Enzyme-Linked Immunosorbent Assay (ELISA) [14], and Luciferase Complementation Assay (LCA) [15], are often hindered by technical complexity and limited analytical scope. Surface Plasmon Resonance (SPR), however, is a cutting-edge analytical approach that exploits SPR to monitor refractive index changes resulting from protein–ligand binding, thereby enabling the precise determination of kinetic parameters and receptor affinities. SPR, with its label-free operation, exceptional sensitivity, real-time monitoring capabilities, low sample consumption, streamlined processing, and high-throughput compatibility, surpasses traditional techniques, making it an indispensable tool in receptor affinity research. The application of SPR technology in the field of drug research cannot be underestimated [16], and the use of SPR technology to determine the CB1 receptor affinity of four SCs has been reported, and the consistency of the results with those of radioisotope-labeled receptor binding assays has also been verified [17]. When utilizing SPR technology to assess the receptor affinity of SCs, one can choose to immobilize relevant antibodies (e.g., designed according to JWH-018) [18]. However, due to the structural similarity and diversity of SCs, the cross-reactivity of antibodies often leads to inaccurate results, and new antibodies may need to be designed for novel compounds, which costs a lot [14]. Directly immobilizing the CB1 receptor offers a more broad-spectrum detection approach, which can better address the rapid structural modifications characteristic. Therefore, in this paper, we chose to immobilize the CB1 receptor proteins onto the chip in order to assess the affinity of five indole-based SCs (JWH-018, AMB-4en-PICA, FDU-PB-22, MAM-2201 and STS-35) and five indazole-based SCs (MDMB-4en-PINACA, 5F-MDMB-PINACA, AB-CHMINACA, 5F-akb-4 and FUB-AKB-48), analyze the structure–affinity relationship, and compare the results with those obtained from conventional assays reported in related reports.

## 2. Results

### 2.1. Ligand Immobilization

**Figure 1:**
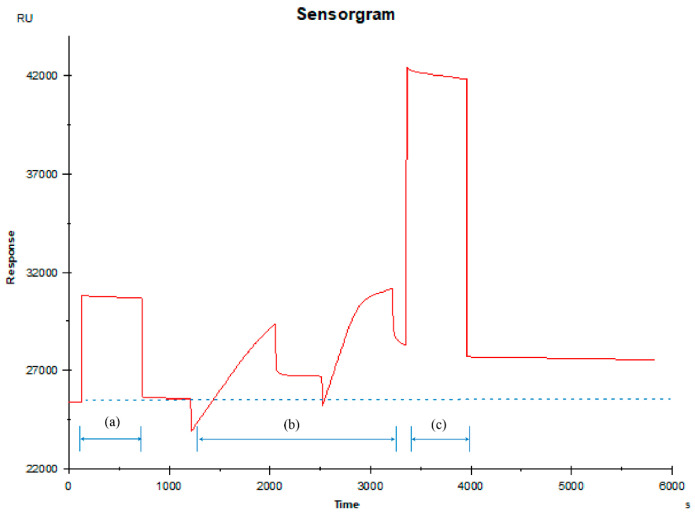
Real-time monitoring of manually coupled proteins (**a**) activation; (**b**) coupling; (**c**) blocking.

The coupling situation of the proteins has an effect on the interaction. Figure 1 showed the real-time dynamic process of CB1 protein immobilization on the surface of the CM5 chip. The process demonstrated three characteristic phases: (a) Initial activation of the CM5 chip surface carboxyl groups was confirmed by a 100–200 response unit (RU) increase following the injection of the NHS/EDC mixture; (b) coupling of CB1 receptor proteins resulted in a substantial increase, verifying amine-mediated conjugation to the activated surface; (c) final blocking with ethanolamine hydrochloride effectively quenched remaining reactive groups, achieving stable baseline stabilization at the immobilization level of 2500 RU, simultaneously minimizing non-specific binding interactions.

### 2.2. Sample Detection

**Figure 2:**
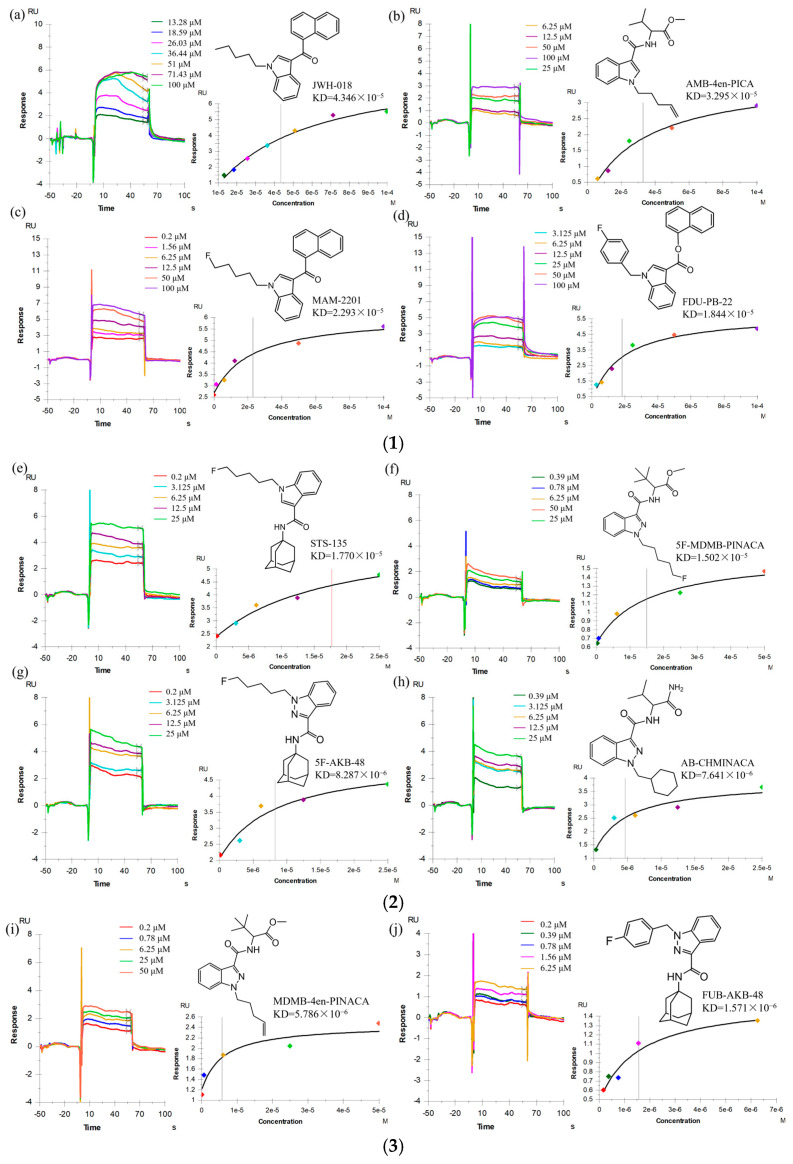
(**1**) Real-time binding sensing plots and affinity profiles of (**a**) JWH-018; (**b**) AMB-4EN-PICA; (**c**) MAM-2201; and (**d**) FDU-PB-22. (**2**) Real-time binding sensing plots and affinity profiles of (**e**) STS-135; (**f**) 5F-MDMB-PINACA; (**g**) 5F-AKB-48; and (**h**) AB-CHMINACA. (**3**) Real-time binding sensing plots and affinity profiles of (**i**) MDMB-4en-PINACA and (**j**) FUB-AKB-48. In the binding and fitting plots, each color represents the corresponding sample concentration. The vertical dashed line in the fitted curve marks the KD values, representing the analyte concentration at which 50% of the binding sites are occupied.

The data were processed using Biacore T200 result analysis software (Biacore T200 Evaluation Software, V3.2) to obtain real-time signal curves of the binding of 10 SC to CB1 receptor proteins, fit affinity curves (Figure 2) and calculate affinity KD values (Table 1). The signal curves encompassed the entire dynamic process of binding and dissociation, showing that all the SCs tested in this experiment exhibited fast binding and fast dissociation kinetics with the CB1 receptor. Affinity fitting results showed differences in the binding affinity of the 10 SCs to the CB1 receptor. The measured KD values showed that FUB-AKB-48 had the highest binding affinity (KD = 1.571 × 10^−6^ M), whereas JWH-018 had the lowest binding affinity (KD = 4.346 × 10^−5^ M), and indazole-based compounds generally exhibited higher binding affinities compared to their indole-based counterparts (unpaired *t*-test, *p* < 0.01). As shown in Table 1, the KD values of indazole-based SCs were all smaller than those of indole-based SCs. For example, STS-135 (KD = 1.770 × 10^−5^ M) and 5F-AKB-48 (KD = 8.287 × 10^−6^ M) share the same head, tail, and linker groups, while the substitution of the parent core from indole to indazole resulted in a 50% reduction in the KD value, indicating a stronger affinity for CB1. Then, the tail and linker groups of AMB-4en-PICA (KD = 3.295 × 10^−5^ M) are identical to those of MDMB-4en-PINACA (KD = 5.786 × 10^−6^ M), with only one methyl group difference in the head, and the most significant distinction lies in the parent core: the indazole ring in MDMB-4en-PINACA resulted in its KD value being only one-sixth of that of AMB-4en-PICA, showing six-fold higher affinity.

Furthermore, the impact of head group modifications on binding affinity was quantitatively assessed through a comparative analysis of structural analogs. Replacement of 5-fluoropentyl with p-fluorophenyl head groups consistently enhanced receptor binding, with FDU-PB-22 (p-fluorophenyl, KD = 1.844 × 10^−5^ M) showing improved affinity over MAM-2201 (5-fluoropentyl, KD = 2.293 × 10^−5^ M) and FUB-AKB-48 (p-fluorophenyl, KD = 1.571 × 10^−6^ M) demonstrating substantially stronger binding than 5F-AKB-48 (5-fluoropentyl, KD = 8.287 × 10^−6^ M).

## 3. Discussion

### 3.1. Ligand Immobilization

As illustrated in Figure 1, the CB1 receptor protein demonstrated effective binding to the CM5 chip through an amino coupling reaction, achieving an immobilization level of approximately 2500 RU. This coupling density proves adequate for the current affinity assay, as evidenced by several key characteristics of the protein–small-molecule binding: the response curves exhibited distinct concentration-dependent patterns with clear differentiation and no overlap or crossover between concentrations. All the curves remained stable and flat, without significant fluctuations, and background noise remained negligible, not interfering with data interpretation. The quantitative analysis revealed that the endpoint concentration response value difference (ΔRU) exceeded 1 RU, while the overall response values throughout the experimental concentration range consistently remained above 1 RU. The fitted R_max_ value reached at least 5 RU, indicating sufficient signal intensity for reliable analysis. Furthermore, the calculated KD values from the affinity curves consistently fell within the experimental concentration range, validating the appropriateness of the selected concentration window. All these results collectively confirmed that the achieved coupling amount of 2500 RU provided an optimal platform for conducting robust and reliable affinity measurements in this experimental setup.

### 3.2. Sample Detection

The equilibrium dissociation constant (KD) serves as a quantitative measure of protein–ligand interaction strength, representing the analyte concentration required for half-maximal receptor occupancy. The smaller the KD value, the stronger the affinity between the protein and the small molecule, and vice versa. As shown in the Results section, the substitution of indole with indazole as the parent nucleus tended to cause an increase in affinity, which was consistent with previous reports. Markham et al. [19] suggested that this may be due to the addition of indazole enhancing the steric complementarity between the ligand and the CB1 receptor binding pocket. The presence of an additional nitrogen atom in the indazole core, as compared to its indole counterpart, facilitates more optimal positioning within the receptor’s active site through the formation of additional hydrogen bonds and improved hydrophobic interactions. This structural modification results in enhanced binding stability due to improved three-dimensional congruence with the receptor’s binding cavity, thereby contributing to the observed increase in binding affinity. And Schoeder et al. [20] found by pharmacological assessment that the electron distribution properties of the indazole ring with higher polarity may enhance electrostatic interactions with key residues of the receptor.

Regarding structural modifications of the head group, a frequently employed strategy involves the incorporation of lipophilic moieties, particularly the introduction of 5-fluoropentyl side chains or p-fluorophenyl groups and related cyclic structures. Previous studies have demonstrated that p-fluorophenyl residues serve as optimal bioisosteric replacements, yielding compounds with comparable or enhanced binding affinity relative to their 5-fluoropentyl counterparts. This can be attributed to three distinct yet synergistic molecular mechanisms. First, the planar aromatic structure of p-fluorophenyl exhibits superior steric complementarity with the hydrophobic subpocket of CB1, facilitating optimal van der Waals interactions with key residues. In contrast, the conformational flexibility of the 5-fluoropentyl side chain results in significant entropic penalties during receptor engagement [21]. Second, the para-fluorine substituent engages in energetically favorable orthogonal dipole–dipole interactions with the backbone carbonyl, and the aromatic system participates in stabilizing π-π stacking interactions with His178 and Trp279-molecular contacts that cannot be established by the aliphatic 5-fluoropentyl chain [22]. Our experimental findings aligned with these literature reports.

Through comparisons with selected conventional assays reported in the literature, our findings demonstrated the notable consistency with established methodologies. As summarized in Table 2 and Table 3, the CB1 receptor affinity ranking of the three SCs determined through the GTPγS binding assay [23] was as follows: JWH-018 (Ki = 3.38 nM) < STS-135 (Ki = 1.93 nM) < 5F-AKB-48 (Ki = 0.87 nM). This ranking closely aligned with the results obtained from our SPR assay. Furthermore, the β-arrestin 2 (β-arr2) recruitment assay conducted in HEK293T cell lines [24] yielded comparable results, demonstrating the affinity sequence: JWH-018 (EC_50_ = 36.8 nM) < 5F-MDMB-PINACA (EC_50_ = 0.84 nM) < AB-CHMINACA (EC_50_ = 0.34 nM). These findings from independent experimental approaches consistently corroborated the reliability and reproducibility of our current SPR methodology.

### 3.3. Limitations

Due to organismal complexity, conducting comprehensive in vivo validation through animal experiments is equally essential. Our laboratory also conducted animal experiments to evaluate the in vivo pharmacological effects of SCs, with the assessment results of behavioral pharmacological potency aligning with the affinity results obtained by SPR measurements in this study [25]. SPR affinity assessments revealed that 4F-ABUTINACA exhibited the strongest binding affinity to the CB1 receptor (KD = 2.014 × 10^−5^ M), followed by JWH-018 (KD = 3.929 × 10^−5^ M) and SDB-005 (KD = 5.302 × 10^−5^ M). This affinity ranking was consistent with the pharmacological outcomes: 4F-ABUTINACA demonstrated the highest acute toxicity (LD50 = 11.62 mg/kg), the most pronounced effects in the tetrad test (e.g., hypothermia, motor suppression, and catalepsy), and the strongest reward potential in the conditioned place preference (CPP) test. Similarly, JWH-018 and SDB-005 showed intermediate and weaker effects, respectively, across these assays. Overall, the consistency between the SPR-assessed affinity data and the in vivo pharmacological results highlights the reliability of SPR as a robust method for assessing the receptor binding affinities of SCs.

On the contrary, I. Canazza et al. systematically investigated the pharmacological properties of several SCs, including JWH-018, 5F-ADB-PINACA, AB-FUBINACA, and STS-135 [26,27]. Through competitive binding assays, they quantitatively determined the receptor affinity of these compounds. Subsequently, they conducted comprehensive behavioral assessments in animal models, evaluating the tetanic effect and sensory responses (visual, tactile, and acoustic). Their findings revealed a notable discrepancy between in vitro receptor affinity and in vivo pharmacological effects, highlighting the complexity of pharmacological responses in living organisms. This inconsistency could be attributed to methodological limitations and various physiological factors, particularly the pharmacokinetic processes encompassing absorption, distribution, metabolism, and excretion (ADME). Consequently, while the study on in vitro receptor affinity serves as a valuable preliminary screening tool for predicting the pharmacological potential of novel compounds, in vivo animal experiments must be complemented in order to obtain a comprehensive understanding of pharmacological efficacy and safety profiles. Future research should address these limitations by supplementing it with animal experiments, integrating SPR with in vivo models, and condcucting systematic pharmacokinetic studies to provide a more comprehensive understanding of the pharmacology of SCs.

Second, it is crucial to acknowledge that experimental outcomes could be significantly influenced by various factors, including the choice of experimental methods, variations in cell lines even when employing the same protocol, and differences in concentration settings and manipulations. To ensure methodological consistency and reliability in our comparative analysis of affinity ordering, we only utilized the data from a single research report conducted by the same investigator. However, this approach might limit the generalizability of our findings.

Additionally, the structural diversity and rapid evolution of SCs pose challenges in extrapolating our results to newly emerging analogs. In the future, we need to test the CB1 receptor affinity of more SCs to derive a more comprehensive and generalized structure–affinity relationship based on the chemical structure.

## 4. Materials and Methods

### 4.1. Experimental Reagents and Instruments

CB1 protein (300 μg/mL, prepared and characterized by China Pharmaceutical University), analytically pure dimethyl sulfoxide (DMSO, Sigma-Aldrich, St. Louis, MO, USA), s-series CM5 chip (cytiva, Marlborough, MA, USA; Made in Sweden), 10 × PBS-P buffer (cytiva, Marlborough, MA, USA; Made in Sweden), amino-coupling kit (cytiva, Marlborough, MA, USA; Made in Sweden), and sodium acetate solution (10 mM, pH = 4.0, 4.5, 5.0, 5.5, cytiva, Marlborough, MA, USA; Made in Sweden), 96-well plate (cytiva, Marlborough, MA, USA; Made in Sweden).

**Figure 3:**
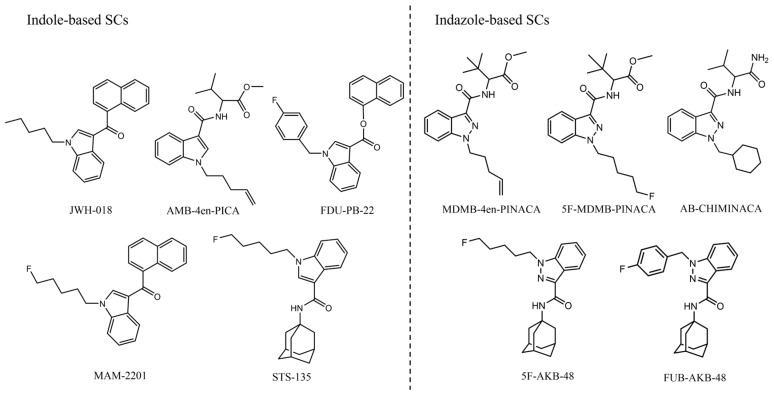
Chemical structures of 10 SCs.

JWH-018 (purity >99%), AMB-4en-PICA (purity > 98%), FDU-PB-22 (purity = 89.5%), MAM-2201 (purity ≥ 98%), STS-135 (purity > 98%), AB-CHMINACA (purity > 98%), 5F-MDMB-PINACA (purity > 98%), MDMB-4en-PINACA (purity > 99%), 5F-AKB-48 (purity > 99%), and FUB-AKB-48 (purity > 99%) were all supplied by the Anti-Drug Intelligence Technology Center of the Ministry of Public Security. The chemical structure formulas are as follows (Figure 3).

Ultrapure water meter (Nanjing Miaoyi Electronic Technology Co., Ltd., Nanjing, JS, China); 3K15 high-speed freezing centrifuge (Sigma, Osterode am Harz, NI, Germany); Biacore T200 biomolecular interaction analyzer (cytiva, Marlborough, MA, USA; manufactured in Sweden).

### 4.2. Ligand Immobilization

The ligand immobilization was conducted in four steps: ligand pre-enrichment, activation, coupling, and blocking. To prepare the 1.05× PBS-P buffer, it was diluted from the 10× PBS-P buffer. For ligand pre-enrichment, the Biacore T200 (cytiva, Marlborough, MA, USA; manufactured in Sweden) pre-enrichment program was employed. The 300 μg/mL protein stock solution was diluted to a final concentration of 15 μg/mL using 10 mM sodium acetate solutions at pH 4.0, 4.5, 5.0, and 5.5, respectively. The ligand was flowed over the surface of a blank chip at a flow rate of 30 μL/min to assess the effect of electrostatic adsorption. Based on the response difference and slope, the optimal coupling pH of 4.0 was selected. For activation, the Biacore T200 manual coupling program was used. The carboxylic acid groups on the chip surface were activated by injecting a 1:1 mixture of EDC and NHS from the amino-coupling kit at a flow rate of 10 μL/min for 900 s. During the coupling step, the 300 μg/mL protein stock solution was diluted 10-fold to 30 μg/mL using sodium acetate at pH 4.0. The diluted protein solution was injected at a flow rate of 10 μL/min for 900 s in two separate injections, allowing the amino groups on the ligand to react with the activated carboxylic acid groups on the chip surface, thereby immobilizing the ligand. Finally, for blocking, the unreacted activated carboxyl groups on the chip surface were blocked by injecting 1 M ethanolamine solution at a flow rate of 10 μL/min for 900 s.

### 4.3. Sample Detection

The buffer used in the sample detection was 1.05× PBS-P, containing 5% DMSO. For sample preparation, it was essential to maintain consistent DMSO concentrations between the samples and buffer throughout the process. First, we dissolved the drug in DMSO to prepare a 2 mM stock solution. The stock solution was diluted 20-fold with 1.05× PBS-P buffer to obtain a 100 μM solution as the highest sample concentration. Serial dilutions (e.g., 100, 50, 25, 12.5, 6.25 μM, etc.) were performed as needed. The dilution factor and maximum concentration were adjusted based on the strength of the interaction between the sample and the protein. Specifically, an initial broad concentration range (e.g., 0.1–100 μM) was tested to assess the binding behavior. The final concentration range was then optimized based on real-time sensorgram analysis: (1) if saturation was achieved at lower concentrations, the range was shifted downward to better characterize the equilibrium phase; (2) if no saturation was observed, the upper concentration was increased until the response curve plateaued. A duplicate concentration and a 0 concentration (blank) were included as controls. A 50% DMSO was prepared as a needle wash solution to clean residual small molecules from the tubing.

For solvent correction, eight solvent correction points were established, with correction cycles performed at the beginning, end, and every 30 samples during the experiment. The correction range was set from −500 to 1000 RU to account for the influence of DMSO on response values. In the multi-cycle kinetic assay, after baseline stabilization, sample injections were initiated. Each injection cycle was followed by a wash step with 50% DMSO to ensure the independent analysis of each concentration. The experiment was conducted at 25 °C with an association time of 60 s, a dissociation time of 120 s, and a flow rate of 30 μL/min. The system continuously monitored and recorded the sensorgram of the interaction between the sample and the CB1 protein in real time. Double referencing and volume exclusion methods were applied to subtract background signals from the blank channel and solvent effects, with data analysis performed using the affinity model in the Biacore T200 evaluation software (Biacore T200 Evaluation Software, V3.2).

Based on the Langmuir model, the interaction between small molecules (analytes) and proteins was assumed to follow a one-stage binding reaction. The dissociation equilibrium constant (KD) was derived from the experimental concentration–response values, while the maximum binding response (R_max_) was also fitted using the analysis software.

The binding kinetics were analyzed using the 1:1 Langmuir interaction model [28]:

A+B⇌AB

The reaction is governed by the association rate constant (k_a_) and the dissociation rate constant (k_d_):

$$k_{a}[A][B]=k_{d}[AB]$$

KD is defined as

$$KD=\frac{\left[A][B\right]}{\left[AB\right]}=\frac{k_{d}}{k_{a}}$$

[A] is the analyte concentration, [B] is the concentration of unbound CB1 receptor protein on the chip surface, and [AB] is the concentration of the bound complex.

When AB binding reaches saturation, the complex concentration is [AB]_max_ at this point:

$${\left[AB\right]}_{max}=[B]\;KD=\frac{\left[A][B\right]}{{\left[AB\right]}_{max}}=[A]$$

When the reaction reaches a steady state, the binding and dissociation processes reach equilibrium, and the complex concentration [AB] reaches a constant value [AB]_t_, the concentration of unbound protein ([B]) is given by: [B] = [AB]_max_ − [AB]_t_. Then,

$$KD=\frac{[A]{\left[AB\right]}_{max}\;}{{\left[AB\right]}_{t}}-[A]\;[{AB]}_{t}=\frac{[A]{\left[AB\right]}_{max}\;}{[A]+KD}$$

When the analyte concentration [A] equals KD, the concentration of the bound complex ([AB]_t_) is half of the maximum binding capacity ([AB]_max_):

$$[A]=KD\;{\left[AB\right]}_{t}=0.5\times {\left[AB\right]}_{max}$$

Thus, KD represents the analyte concentration at which half of the binding sites on the protein are occupied. A lower KD value indicates a stronger binding affinity between the analyte and the protein.

## 5. Conclusions

In summary, this study successfully employed SPR technology to determine the CB1 receptor affinity of 10 SCs, including both indole-based and indazole-based compounds, containing novel SCs of which affinity data were previously unavailable. The SPR method enabled efficient and precise measurement of interaction parameters, facilitating a detailed analysis of structure–affinity relationships. Our findings revealed that indazole-based SCs exhibit stronger CB1 receptor affinity compared to their indole counterparts, and the p-fluorophenyl head structure demonstrated comparable or enhanced affinity relative to the 5-fluoropentyl group. In addition, the affinity rankings obtained through SPR were consistent with those derived from conventional detection methods, further validating the reliability of our approach. These results underscored the applicability of SPR technology for assessing synthetic cannabinoid receptor affinity, highlighting its ability to discern affinity differences among structurally diverse compounds. This study not only confirmed the feasibility of SPR in drug affinity detection but also expanded its potential applications in pharmaceutical research. As a convenient, highly sensitive, and high-throughput analytical tool, SPR is poised to become an indispensable technique for drug–receptor affinity determination, offering significant advantages over traditional methods in terms of efficiency and precision.

## Figures and Tables

**Table 1:** KD values of the 10 SCs.

Classification	Substance	KD Value (M)
Indole-based	JWH-018	4.346 × 10^−5^
	AMB-4en-PICA	3.295 × 10^−5^
	MAM-2201	2.293 × 10^−5^
	FDU-PB-22	1.844 × 10^−5^
	STS-135	1.770 × 10^−5^
Indazole-based	5F-MDMB-PINACA	1.502 × 10^−5^
	5F-AKB-48	8.287 × 10^−6^
	AB-CHMINACA	7.641 × 10^−6^
	MDMB-4en-PINAC	5.786 × 10^−6^
	FUB-AKB-48	1.571 × 10^−6^

**Table 2:** Comparison of SPR results with those of GTPγS binding experiments.

Substance	KD	GTPγS Binding Assay Ki Value (nM)	Literature
JWH-018	4.346 × 10^−5^	3.38	[23]
STS-135	1.770 × 10^−5^	1.93	
5F-AKB-48	1.431 × 10^−5^	0.87	

**Table 3:** Comparison of SPR results with that of βarr2 recruitment experiments.

Substance	KD	βarr2 Recruitment Assay EC_50_ Values (nM)	Literature
JWH-018	4.346 × 10^−5^	36.8	[24]
5F-MDMB-PINACA	1.502 × 10^−5^	0.84	
AB-CHMINACA	7.641 × 10^−6^	0.34	

## Data Availability

Data are contained within the article.
